# Plasticity and the Transition from Physical Gels to Yielding Liquids

**DOI:** 10.3390/gels12080687

**Published:** 2026-08-03

**Authors:** Alexander Ya. Malkin, Svetlana R. Derkach, Vlada V. Bordiyan

**Affiliations:** 1 Laboratory of Polymer Rheology, A.V. Topchiev Institute of Petrochemical Synthesis, Russian Academy of Sciences, 29 Leninskii Prospect, Moscow 119991, Russia; 2 Natural Science and Technologies Institute, Murmansk Arctic University, 13 Sportivnaya St., Murmansk 183010, Russia; derkachsr@mauniver.ru (S.R.D.); bordiyanvv@mauniver.ru (V.V.B.)

**Keywords:** gel, rheology, yielding liquids, plasticity, viscosity

## Abstract

This study examines the possibility of plastic deformation in low-modulus gels and yielding liquids. The model systems were a gelatin-sodium alginate hydrogel and the same gel filled with nanoscale zinc oxide particles. The experiments involved short- and long-term observations of deformation development under a prescribed shear stress, followed by stress removal and monitoring of deformation recovery. The initial hydrogel is a typical soft-matter system with an elastic modulus of 63 Pa. At low stresses, residual deformations were observed in addition to elastic deformations; these residual deformations reached up to approximately one half of the total deformation. They appeared instantaneously, depended on the applied stress, and did not change during long-term observation. This behavior is characteristic of plastic deformation. The incorporation of 5% dispersed ZnO nanoparticles converted the gel into a yielding liquid. This transition is attributed to partial disruption of the physical network, as evidenced by a sharp decrease in the elastic modulus to 18 Pa and by comparison of the FTIR spectra of the unfilled gel and the nanoparticle-modified gel. The yield stress of the yielding liquid was 7 Pa. However, at stresses below this value, while the material remained in a gel-like state, steady-state flow with a very high viscosity, at the order of 10^5^–10^6^ Pa s, was detected. After the yield point was exceeded, steady-state flow with a much lower viscosity, at the order of 3 Pa s, occurred, as is characteristic of conventional liquids containing a solid filler. Nevertheless, a small fraction of plastic deformation was still observed. Thus, the experimental results show that physical gels can behave as elastic-plastic media and that yielding liquids may flow below the yield point with very high viscosity. All existing models of the mechanical behavior of gels represent various combinations of viscous and elastic elements, to which, for yielding liquids, a slider is added that begins to slide after overcoming static friction. This element models the yield point. However, no model includes plasticity as an independent mechanical phenomenon. The phenomenon of plasticity should therefore be taken into account when developing rheological models of yielding liquids.

## 1. Introduction

The rheology of gels and yielding liquids is a broad field of study that began with the pioneering work of Bingham, who discovered the existence of a yield point in suspensions and used the term “plasticity” to describe viscous flow at stresses exceeding this yield point [1]. Thus, in rheology, flow at stresses above the yield point is traditionally defined as a region of plastic behavior. Accordingly, many studies describe yielding liquids in terms of viscoplasticity. This concept applies to a wide range of media which, following de Gennes’ classification, are classified as soft matter because of their low elastic modulus [2]. Such rheological behavior is typical of many real materials, including colloids, non-colloidal suspensions, emulsions, polymeric systems, food products, and natural phenomena such as landslides and avalanches. These materials occupy an intermediate position between ordinary liquids, which flow under arbitrarily small stresses, and gels.

It is important, however, to distinguish between gels and yielding liquids. Gels are media in which a network of structural bonds is formed in a liquid phase as a result of incomplete phase separation in a multicomponent mixture. This network must be sufficiently strong to prevent flow under external loading and, like any solid, to disintegrate when the load exceeds the ultimate strength of the material. Yielding liquids, by contrast, can exist in two rheological states: at low stresses they behave as solids or gel-like materials, whereas above a certain critical stress they flow as liquids [3,4]. Nevertheless, especially in applied research, highly concentrated and highly viscous polymer solutions are often referred to as gels. It should also be noted that polymer solutions are single-phase materials, although relatively large and long-lived heterophase fluctuations caused by intermolecular interactions may occur. Gels, in contrast, are heterophase systems in which one component forms a continuous percolated structure within a liquid matrix.

Around the same time as Bingham’s work, von Mises published a study that also introduced the concept of plasticity, but in relation to metals [5]. The only common feature of the two cases was that both involved large deformations. Their physical meaning, however, was different. For elastic metals, irreversible deformations were considered, but these deformations were not the same as flow. This interpretation of plasticity became standard in solid mechanics [6,7]. Plastic deformation of solids, both crystalline and amorphous, develops after a certain stress threshold is reached, marking the transition from reversible elastic deformation to irreversible plastic deformation. Thus, as in yielding liquids, a transition from one type of rheological behavior to another occurs. However, the nature of these transitions is fundamentally different. In yielding liquids, the transition is from solid-like behavior to liquid-like flow, whereas in solids it is a solid–solid transition. In both cases, the transition results in irreversible deformation, but the physical nature of the deformation is different. In yielding liquids, flow leads to an unlimited increase in deformation with time under an applied stress. In solids, plastic deformation is limited and depends on stress rather than time. Since both transitions involve structural changes, certain analogies can be drawn between them, including time- or rate-dependent effects [8], thixotropic transformations and aging [9,10], and a formally equivalent transition criterion expressed through the work of shear stresses under three-dimensional loading [11]. Nevertheless, these phenomena remain fundamentally different, as discussed in [12]. In that publication, plasticity was developed as a component of soft-matter deformation. The mechanism of plasticity depends on the phase state of the material. In crystalline bodies such as metals, plasticity is associated with phase transformations in the crystal lattice or with slip along dislocations [13,14,15]. Plasticity is also known in amorphous materials, including soft-matter systems such as highly filled suspensions [16], fuel-oil-impregnated sand [17], and partially melted ultra-high-molecular-weight polyethylene [18].

The present work develops these studies and the general concept proposed in [12]. Its aim is to answer the following questions: Are plastic deformations possible in typical gels, and how does the transition from elastic-plastic deformation in the gel-like state to viscous flow occur in yielding liquids? To address these questions, we performed systematic studies on two typical soft-matter systems: an unfilled hydrogel and the same gel filled with a hard solid filler.

In essence, we seek to clarify how general the phenomenon of plastic deformation is in different soft-matter systems. We believe that answering this question is important for understanding soft-matter rheology and for constructing rheological models of solid-like soft matter more accurately, moving beyond the view of such materials as purely viscoelastic or elastic-flow media.

## 2. Results and Discussion

### 2.1. Microstructure of the Samples

**Figure 1:**
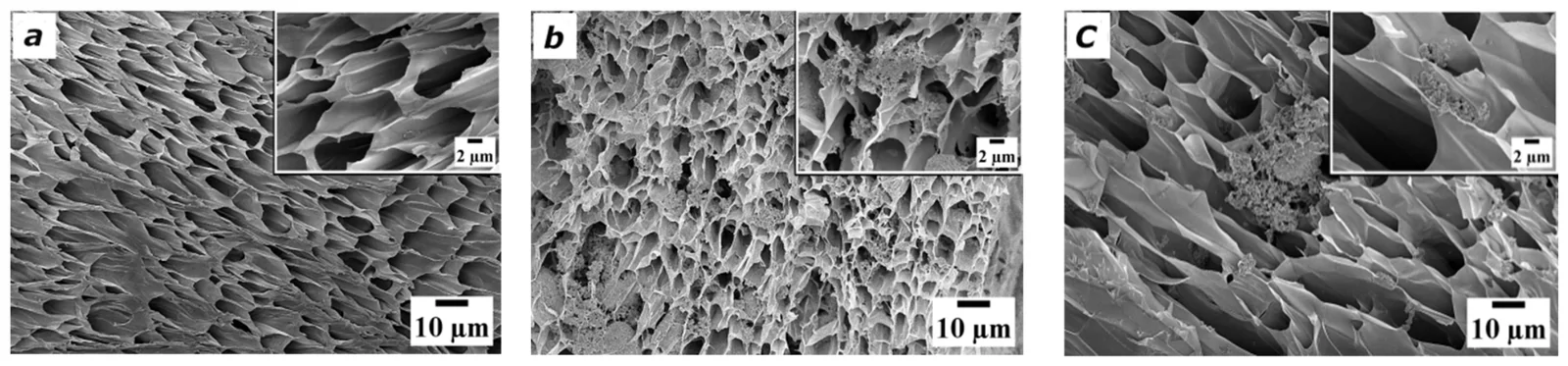
SEM images of the gelatin-sodium alginate complex gel GSA (**a**) and the same gel containing 2% ZnO nanoparticles, GSA+2ZnO (**b**), and 5% ZnO nanoparticles, GSA+5ZnO (**c**).

Micrographs of the samples are presented in Figure 1.

**Figure 2:**
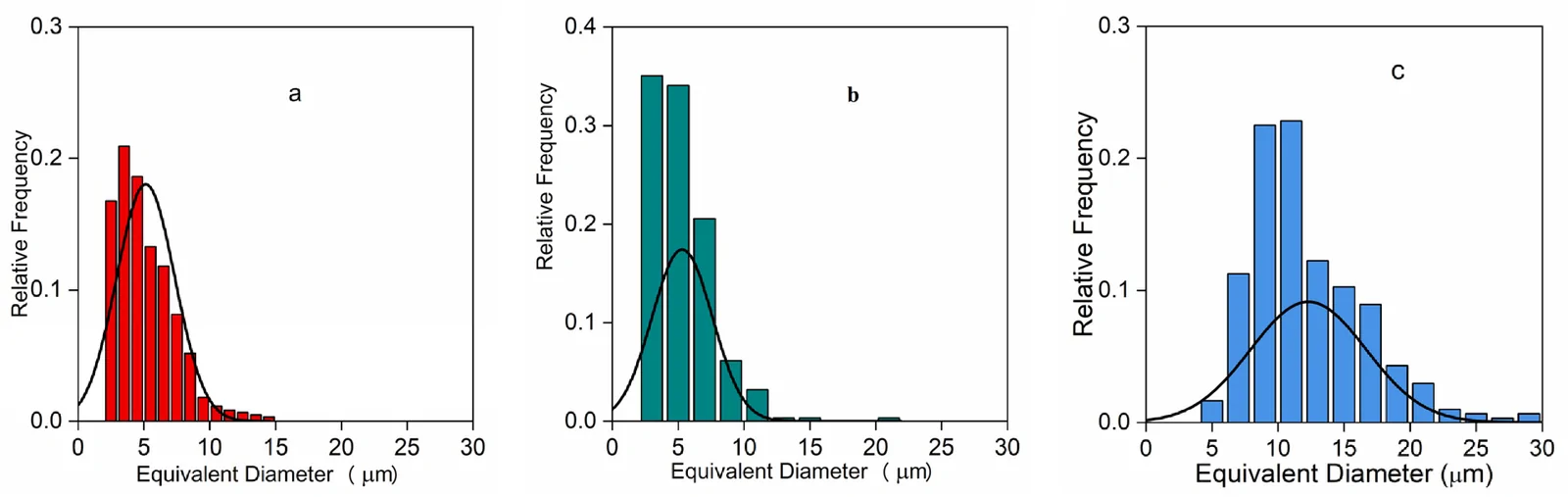
Histograms of the pore (cell) size distribution for the GSA gel (**a**) and the corresponding compositions containing 2% ZnO (**b**) and 5% ZnO (**c**).

Microstructural analysis of the gelatin-sodium alginate hydrogels showed that the samples have a layered architecture with elongated pore-like cells (Figure 2a). The introduction of zinc oxide nanoparticles led to the formation of nanoscale agglomerates on the surface of the polymer matrix. At a concentration of 2%, the agglomerates were irregular in shape, had a spongy structure, and were locally distributed; overall, the structure remained close to that of the initial gel (Figure 2b). Increasing the filler content to 5% substantially increased the number of particle agglomerates and the surface coverage. The porosity also visibly increased. At the same time, the particle distribution became denser and more uniform (Figure 2c). The layered architecture of the polymer matrix was preserved in all three samples. Histograms of the cell-size distribution for the three samples are shown in Figure 2.

**Figure 3:**
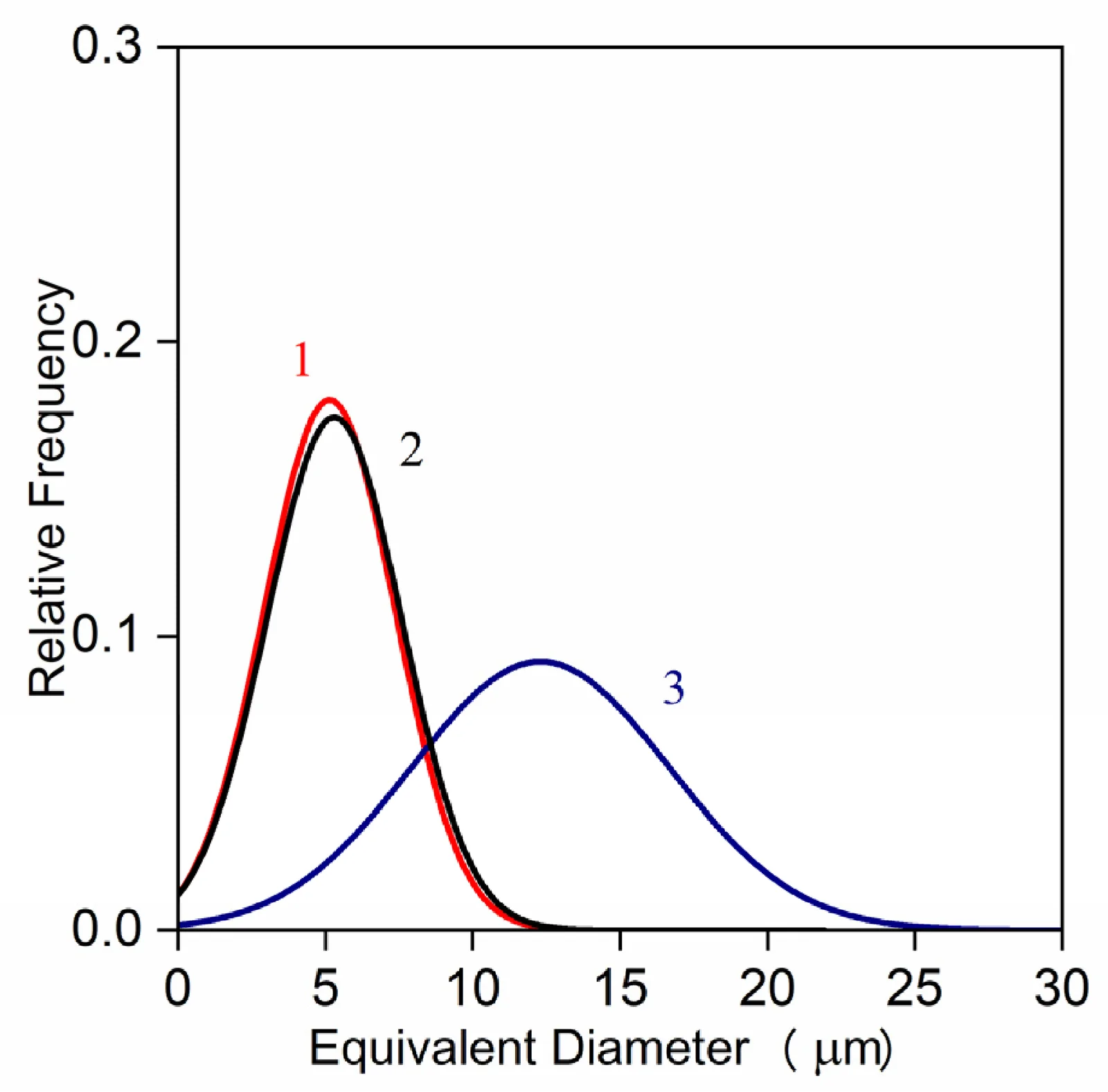
Pore-size distributions for the GSA gel (1) and the corresponding compositions containing 2% ZnO (2) and 5% ZnO (3).

The original gel without filler was characterized by an average pore size of 5.1 ± 2.2 μm and a narrow distribution (Figure 2a). The introduction of 2% ZnO practically did not change the average pore size, which shifted only slightly to 5.3 ± 2.3 μm (Figure 2b). In contrast, the introduction of 5% ZnO resulted in a marked increase in the average pore size to 12.3 ± 4.4 μm (Figure 2c). A comparison of the pore-size distributions for the three samples is presented in Figure 3.

Thus, the introduction of 2% filler only slightly changes the gel structure, whereas the introduction of 5% filler produces a significantly looser material structure.

### 2.2. Theoretical Basis of the Rheological Measurements

The aim of the experiments was to evaluate the rheological behavior of the gel and yielding liquids under creep and elastic recovery conditions during short- and long-term loading, and to clarify the role of the nanodispersed filler in these experiments.

The development of deformation γ in a viscoelastic material subjected to an arbitrary stress history σ, within the linear region of mechanical behavior and within the framework of the Boltzmann-Volterra model, is described by the equation

(1) $$\gamma (t)=\int_{0}^{t}\frac{d\sigma }{dt}\left[J_{0}+\psi (t)+\frac{t}{\eta }\right]dt$$

where J_0_ is the equilibrium or instantaneous compliance, including elastic and residual components; η is the viscosity; t is time; and ψ(t) is the creep function, expressed as

(2) $$\psi (t)=\int_{0}^{\infty }J(\lambda )\left(1-e^{-t/\lambda }\right)dt$$

where λ is a retardation time and J(λ) is the weight function representing the spectrum of retardation times.

For the solids considered here, J_0_ is the compliance associated with residual deformation, Jres, because Hookean deformation in these viscoelastic media is negligible and η tends to infinity. The limiting elastic deformation at t → ∞ is therefore

(3) $$\gamma_{el}=\sigma \int_{0}^{\infty }J(\lambda )d\lambda$$

The experiment on which this work is based was implemented according to the following protocol:

(4) $$\sigma (t)=\sigma \left(\begin{array}{c} 1\\ 1-\delta \left(t^{*}\right) \end{array}\right.\;\begin{array}{c} t<t^{*}\\ t\geq t^{*} \end{array}$$

where δ(t*) is the delta function and t* is the time corresponding to the transition from loading at stress σ to unloading and the beginning of retardation, i.e., elastic recovery.

Thus, during loading at stress σ, deformations develop according to

(5) γ(t) = σ[J_0_ + ψ(t)]

The product J_0_σ represents the residual deformation, and the maximum plateau deformation reached as a result of creep is therefore

(6) $$\gamma_{plateau}=\sigma \left[J_{0}+\int_{0}^{\infty }J(\lambda )d\gamma \right]=\gamma_{res}+\gamma_{el}$$

During the second stage of the process, i.e., retardation, the change in deformation is described according to Equation (3) as

(7) $$\gamma (t)=\gamma_{plateau}-\sigma \left[J_{0}+\psi (t)\right]$$

The final result of retardation is the residual deformation, determined as

(8) $$\gamma_{res}=\gamma_{plateau}-\sigma \int_{0}^{\infty }J(\lambda )d\lambda =J_{0}\sigma$$

**Figure 4:**
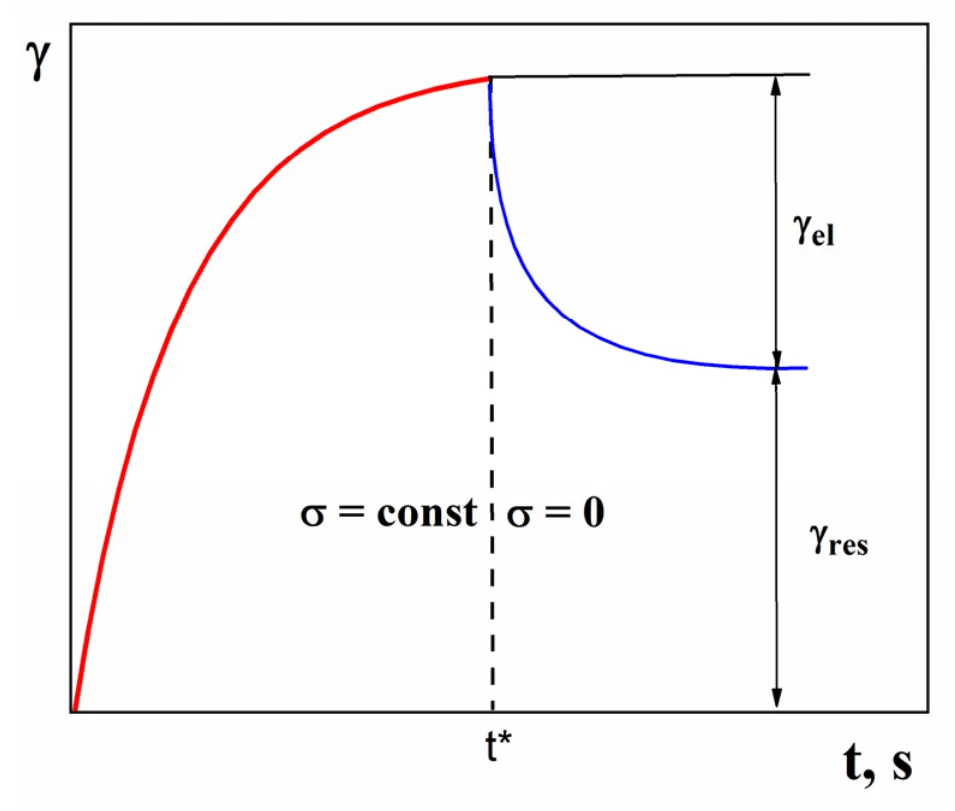
Protocol for measuring rheological quantities used in the subsequent analysis. Red line - loading, blue line-retardation. gamma sub res-residual deformation, gamma sub el-elastic deformation, sigma-stress.

This protocol is illustrated in Figure 4, which shows the experimental scheme used to determine γres and γel.

For viscoelastic liquids, Equation (1) should be written in the following form:

(9) $$\gamma (t)=\int_{0}^{t}\frac{d\sigma }{dt}\left[J_{res}+\psi (t)+\frac{t}{\eta }\right]dt$$

The development of deformation at σ = const is then given by

(10) $$\gamma (t)=\sigma \left(J_{res}+\psi (t)+\frac{t}{\eta }\right)$$

In the limiting case, at sufficiently large t, the last term in this equation dominates, and

(11) $$\frac{\gamma (t)}{\sigma }=\frac{t}{\eta }$$

The viscosity is then obtained from the simple relationship

(12) $$\frac{1}{\eta }=\frac{d\gamma (t)}{\sigma dt}=\frac{\dot{\gamma }}{\sigma }$$

where $\dot{\gamma }$ is the shear rate.

The relationships presented above belong to the linear theory of viscoelasticity. However, Equations (6) and (10) have a more general meaning because they do not explicitly include the creep function, which becomes stress-dependent in the nonlinear case.

In addition, viscoelastic media are characterized by the frequency dependence of the complex elastic modulus G*(ω), which consists of the storage modulus G′(ω) and the loss modulus G″(ω):

(13) $$G^{*}(\omega )=G^{\prime }\left(\omega \right)+iG^{\prime \prime }\left(\omega \right)$$

The ratio between G′ and G″ determines whether the material behaves predominantly as a viscoelastic solid (G′ > G″) or as a viscoelastic liquid (G″ > G′).

The equations of viscoelasticity provide the basis for the following measurement protocol at σ = const (Figure 4).

The first part of the experiment consists of measuring the time-dependent development of deformation, γ(t), under a constant applied shear stress, σ = const. The stress is then removed at time t_0_, and recovery is measured.

The reverse deformation at t > t0 consists of two components: a reversible component, which has the obvious meaning of elastic deformation γel, and an irreversible or residual component, γ_res_. For a viscoelastic liquid, γres should correspond exactly to the deformation associated with flow. If this is the case, there should be a correspondence between the slope of the linear part of the γ(t) curve and the residual deformation γ_res_. To establish such a correlation, we conditionally assume that the slope in the final part of the curve, (dγ/dt) at t → t0, corresponds to the flow deformation rate. The apparent viscosity corresponding to this slope can then be estimated as

(14) $$\eta_{ap}=\frac{\sigma }{{\left(d\gamma /dt\right)}_{t\to t_{0}}}$$

The deformation associated with flow, accumulated over the entire deformation time under the applied stress, can then be estimated as the apparent accumulated “flow” deformation

(15) $$\gamma_{app.fl}={\left(d\gamma /dt\right)}_{t=t_{0}}t_{0}$$

However, this is only an upper estimate. With further deformation, the shear rate may become even lower, and the actual viscosity correspondingly higher.

For a viscoelastic liquid, the residual deformation γ_res_ should correspond exactly to the flow deformation. However, this is far from the case in all systems considered below: γ_res_ > γ_app,fl_, and therefore γres cannot be attributed to flow. An even more important experimental fact is that γ_res_ does not depend on deformation time and therefore cannot be associated with flow. It should therefore be regarded as irreversible plastic deformation in the sense used in solid mechanics.

### 2.3. Linear Viscoelasticity of the Samples

**Figure 5:**
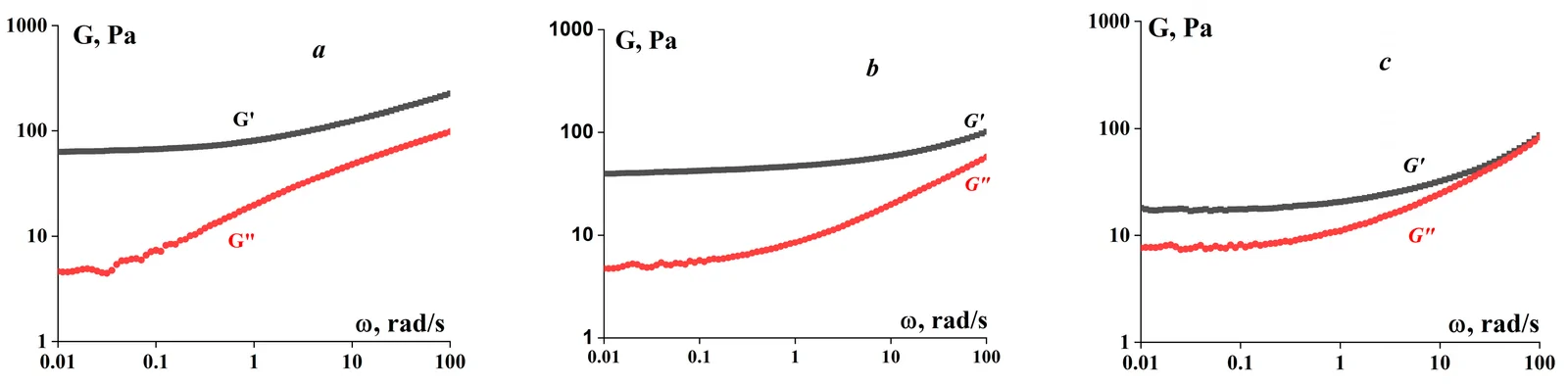
Frequency dependences of the storage modulus G′(ω) and loss modulus G″(ω) for the samples studied: GSA (**a**), GSA+2ZnO (**b**), and GSA+5ZnO (**c**) at 4 °C.

The rheological characteristics of all samples are presented in Figure 5 as frequency dependences of the components of the dynamic modulus in the linear viscoelastic region.

These data indicate that the materials studied are solid-like bodies and, based on their elastic moduli, can be classified as soft matter. Dissipative losses become noticeable at high frequencies, corresponding to the region of fast relaxation, whereas predominantly elastic behavior is observed in the long-time region. The GSA+5ZnO sample differs most strongly from the original GSA sample in the high-frequency region, where viscous losses become comparable to the elastic response.

**Figure 6:**
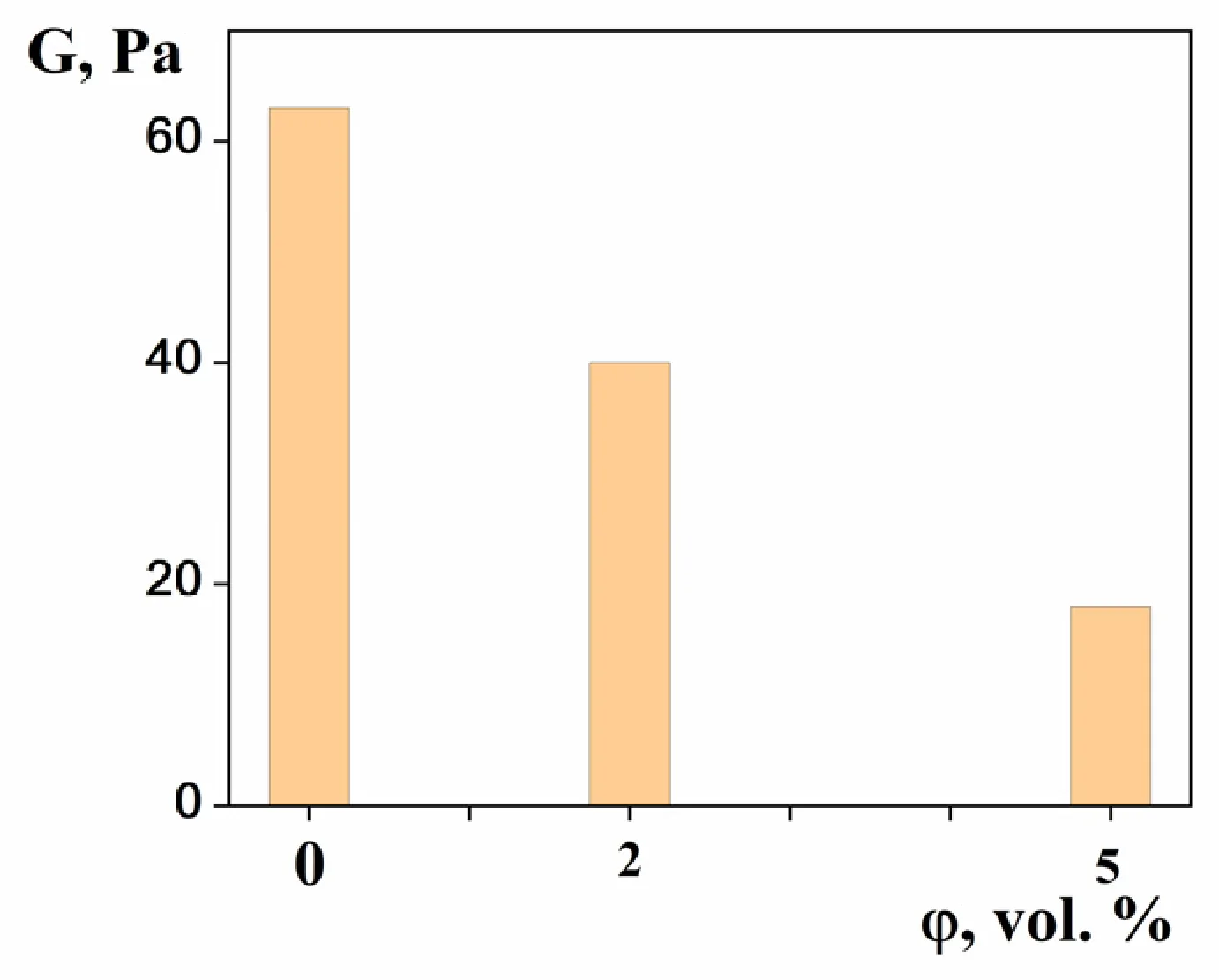
Equilibrium elastic moduli of the GSA gel and ZnO-containing compositions.

It is also interesting to note the unexpected effect of the solid filler on gel elasticity. One might expect the low-frequency elastic modulus, i.e., the equilibrium modulus, to increase with increasing ZnO concentration. In reality, however, the elastic modulus decreases from 63 Pa for GSA to 40 Pa for GSA+2ZnO and only 18 Pa for GSA+5ZnO (Figure 6).

The addition of a filler to elastic media usually leads to an increase in the elastic modulus. A typical example of such behavior of filled gels indicates a very sharp increase in the elastic modulus with increasing filler concentration [19]. In the case under consideration, we have an unexpected reverse effect.

The physical network responsible for the elasticity of the material arises from interactions between the components of the GSA composition. As shown in Figure 3 and Figure 4, filler introduction leads to rarefaction of the structural network; this effect is minor for GSA+2ZnO but substantial for GSA+5ZnO. This explains the decrease in the elastic modulus, which is directly related to the density of the structural network. The physical reason for this phenomenon is that secondary bonds between the gel components are partially replaced by bonds between polar groups in the polymers and ZnO, as confirmed by the IR-spectroscopy data discussed below.

### 2.4. Creep and Recovery

**Figure 7:**
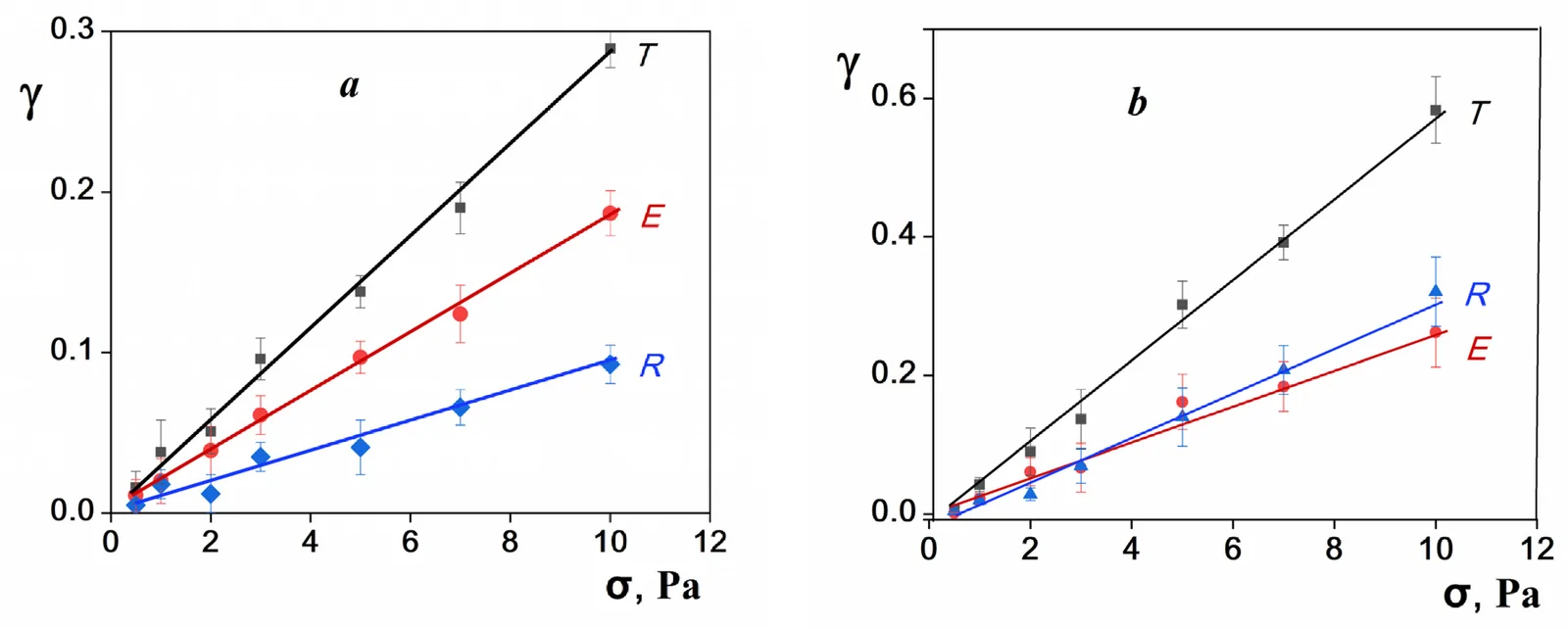
Dependence of deformation components on stress under short-term loading (35 min) for: GSA (**a**) and, GSA+5ZnO (**b**) at 4 °C. T denotes the total deformation, E the elastic component, and R the residual or irreversible component.

Experimental data showing the dependence of deformation on applied constant stress and the subsequent elastic recovery after stress removal are shown in Figure 7.

It can be seen that GSA is a gel capable primarily of elastic deformation. Residual deformations account for approximately one third of the total deformation, and γ_res_ < γ_el_. The situation is different for GSA+5ZnO. As noted above, this gel is softer than GSA, and in this case residual deformations exceed elastic deformations.

Analysis of irreversible deformation according to the scheme described above showed that the conditional apparent viscosity (Equation (14)) is approximately 4 × 10^5^ Pa s in many cases. This is only an estimate, because the measurement results depend on stress and show considerable scatter. In all cases, γ_fl_ did not exceed 0.01, which is approximately 10% of the total observed deformation. Thus, flow, even if present, can be neglected in comparison with the total deformation.

**Figure 8:**
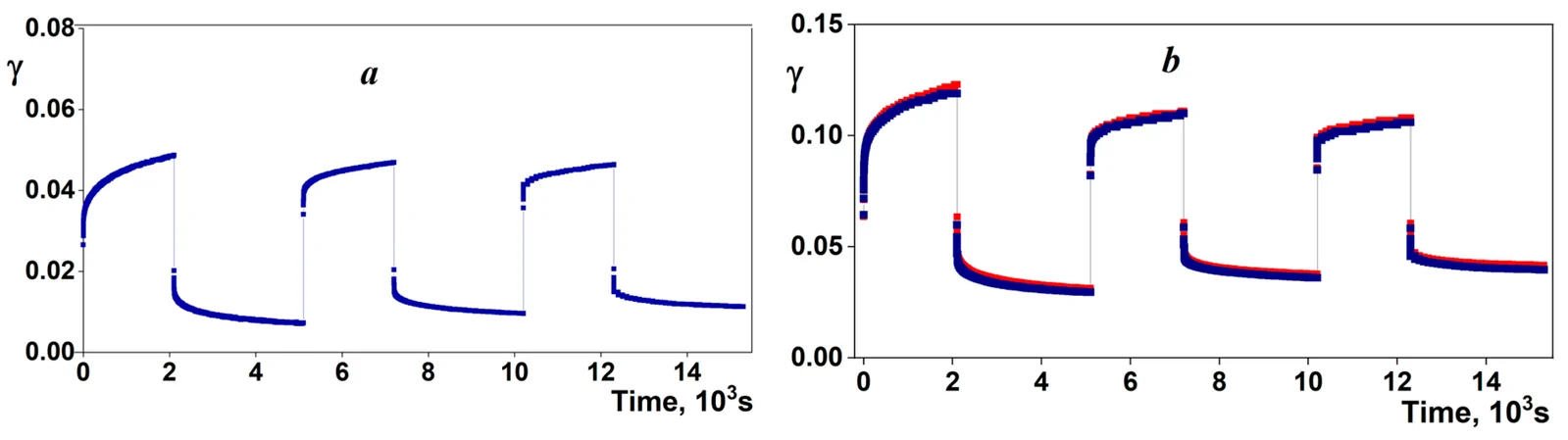
Results of cyclic tests of the GSA sample at stresses of 2 Pa (**a**) and 5 Pa (**b**). The blue and red curves in panel (**b**) illustrate the reproducibility of the experimental data.

The nature of the deformation is clearly visible during repeated loading–unloading cycles. These data are presented in Figure 8 for two stresses, 2 and 5 Pa. The second case also illustrates the reproducibility of the results: the second experiment was performed on a fresh sample prepared several days after the first test. The repeated curves almost exactly reproduce the previous ones, possibly with a slight decrease in total deformation because prior loading somewhat stabilizes the gel structure. These results confirm the elastic-plastic nature of deformation in the GSA gel.

**Figure 9:**
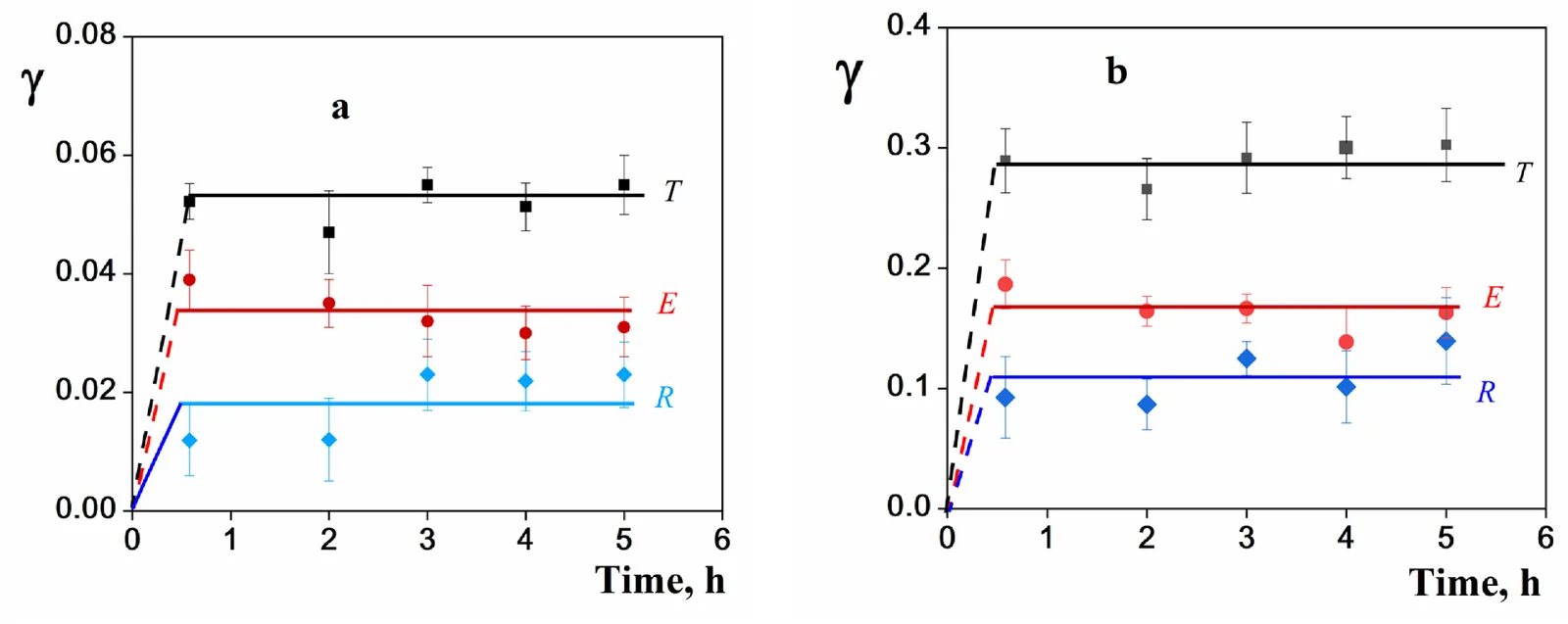
Time dependence of the total deformation components of the unfilled GSA gel under long-term loading for 5 h at stresses of 2 Pa (**a**) and 10 Pa (**b**). T denotes the total deformation, E the elastic component, and R the residual or irreversible component.

**Figure 10:**
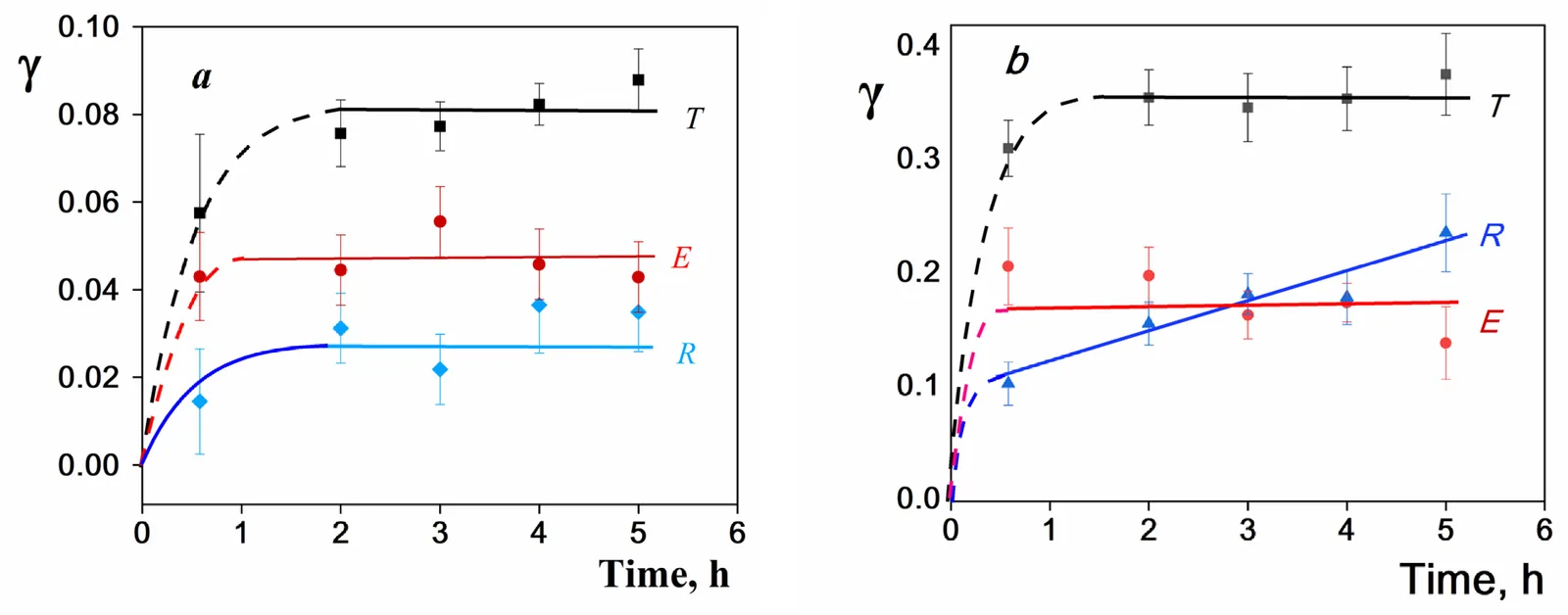
Time dependence of the total deformation components of the GSA+2ZnO sample under long-term loading for 5 h at stresses of 2 Pa (**a**) and 10 Pa (**b**). T denotes the total deformation, E the elastic component, and R the residual or irreversible component.

**Figure 11:**
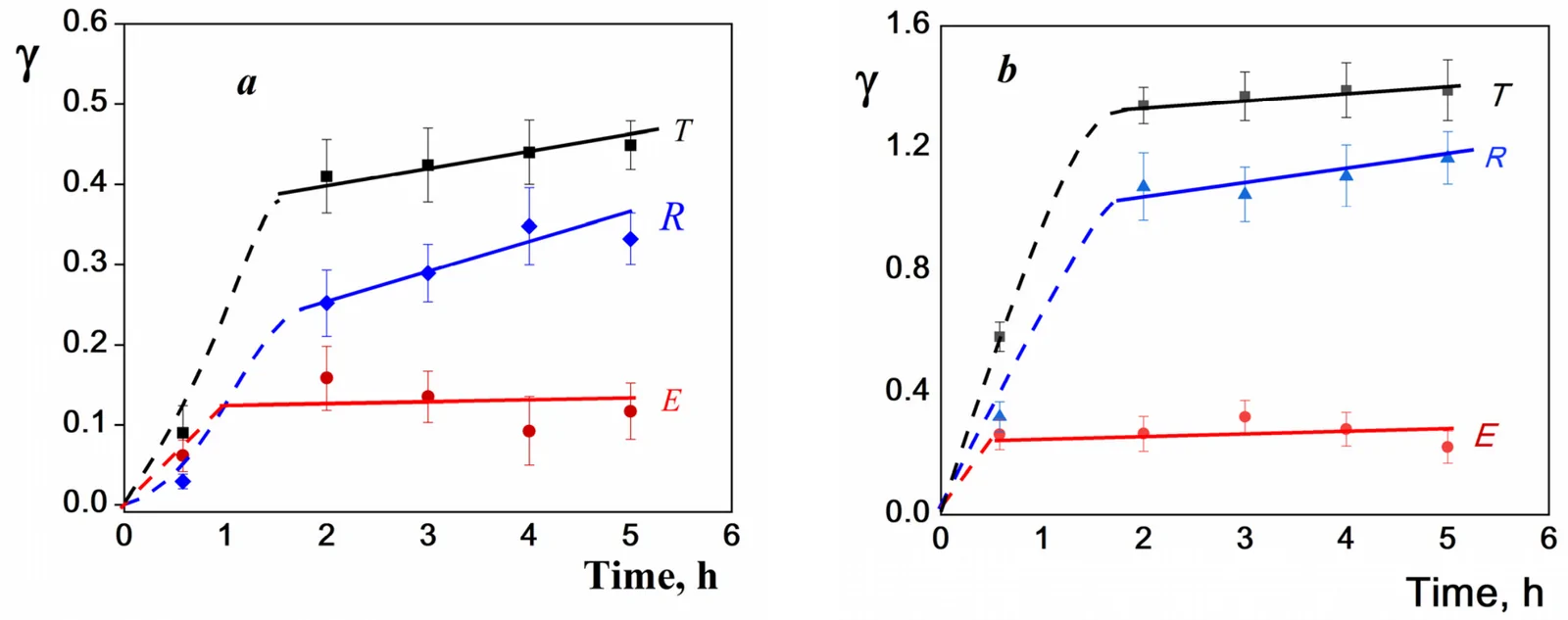
Time dependence of the total deformation components of the GSA+5ZnO sample under long-term loading for 5 h at stresses of 2 Pa (**a**) and 10 Pa (**b**).

We now consider the results of long-term experiments lasting up to 5 h, which were carried out at two stresses, 2 and 10 Pa. The experimental data are presented in Figure 9 for the unfilled GSA gel and in Figure 10 and Figure 11 for the ZnO-filled gels containing 2 and 5% filler, respectively.

T denotes the total deformation, E the elastic component, and R the residual or irreversible component.

The experimental results allow several general conclusions to be drawn regarding the nature of gel deformation. As seen in Figure 10, the GSA sample is a true gel. The observed irreversible deformations are undoubtedly plastic. Their specific feature is that they develop simultaneously and in parallel with elastic deformations. This distinguishes the observed behavior from the elastic-plasticity of solids such as metals, glasses, and ceramics. In classical elastic-plastic media, only elastic deformations occur at low stresses; after a certain threshold is reached, plastic deformations replace them. This threshold is called the yield strength or yield stress and, under a complex stress state, is determined by the work of tangential stresses, i.e., the Huber–Mises–Hencky criterion. Reaching the plasticity threshold is associated with the accumulation of a critical energy responsible for changing the shape, but not the volume, of the body.

At stresses above this threshold, large irreversible deformations develop, and in technological practice it is sometimes said that above the yield point a metal “flows.” Strictly speaking, however, this is not flow but plastic deformation. The fundamental difference between plastic deformation and flow is that, in the plasticity region, there is a one-to-one correspondence between stress and strain that is independent of time, whereas under constant stress flow deformation increases indefinitely with time. Thus, the yield stress in elastic-plastic media reflects a solid–solid transition, in contrast to the yielding phenomenon widely discussed in rheology, where the yield stress corresponds to a solid–liquid transition.

The mechanism of plastic deformation in amorphous media is associated with microstructural heterogeneity, as demonstrated in many studies [16,20]. In such media, free volume remains available for structural elements to move under external stress. The nature of this free volume can differ. In concentrated suspensions, it is associated with heterogeneity in the distribution of solid particles within the matrix. However, this volume is insufficient for flow, because the movement of particles is limited by their trapping between denser structural elements. The physics of this phenomenon is discussed in [21,22]. In gels formed by physical bonds, heterogeneity is expressed in the distribution of these bonds along macromolecular chains. Applied stress may be sufficient to break some of these bonds, which then reform elsewhere, allowing spatial mobility of the macromolecules and thereby producing plasticity in the gel. Preliminary calculations show that the energy supplied by external loading at stresses of approximately 2–10 Pa is sufficient to break a substantial number of intermolecular secondary bonds.

The behavior of the GSA sample is therefore clear: it is a solid-like gel with elastic-plastic properties. The introduction of a solid filler into the gel, however, changes this picture.

This sample exhibits rheological behavior consistent with a yielding liquid. At low stress (Figure 11a, 2 Pa), the sample behaves as a solid-like gel and demonstrates elastic-plastic behavior. At higher stress (Figure 11b, 10 Pa), residual deformation initially increases sharply, as in the unfilled gel, but then continues to increase slowly with time, which should be interpreted as steady-state flow. Thus, filling leads to a transition from a gel to a yielding liquid.

**Figure 12:**
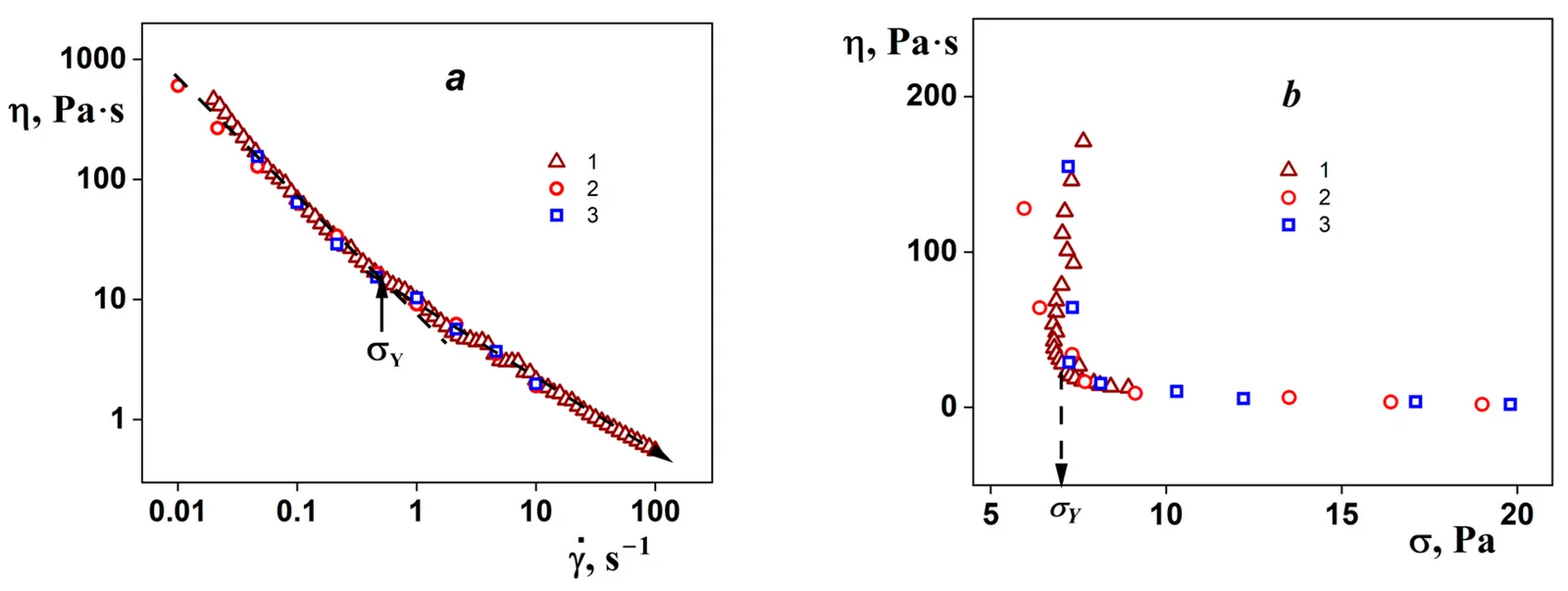
Flow curves of the suspension containing 5% ZnO (GSA+5ZnO) plotted as apparent viscosity versus shear rate (**a**) and apparent viscosity versus shear stress (**b**). The experimental points correspond to different durations of steps at constant shear rate in the shear-rate scanning mode: 1, 60 s; 2, 600 s; and 3, 1200 s.

**Figure 13:**
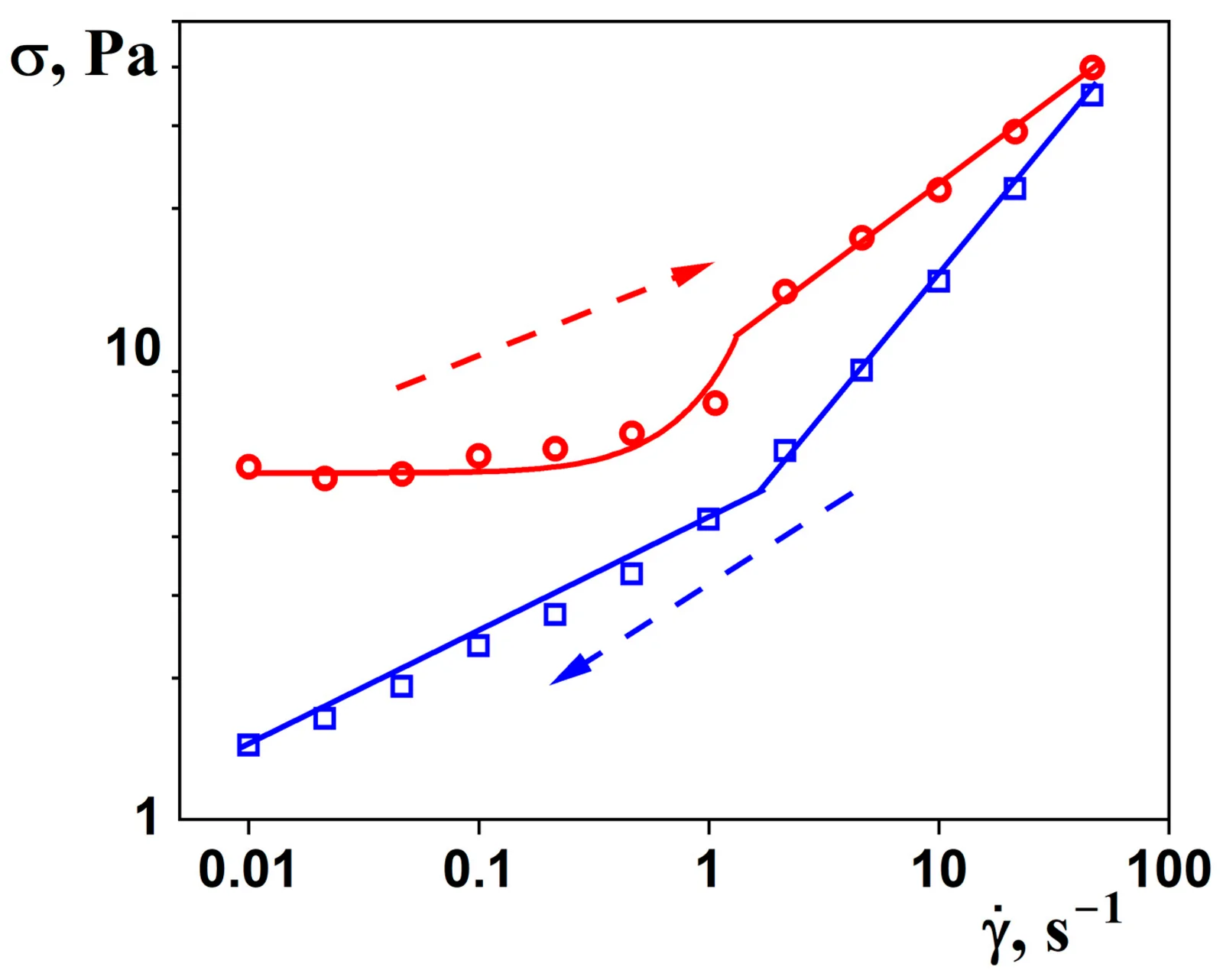
Up-and-down shear-rate scan for the suspension containing 5% ZnO, illustrating thixotropic behavior.

The situation becomes especially interesting for the sample with the higher filler content, GSA+5ZnO (Figure 12). This material is clearly a yielding liquid. As shown below (Figure 13), the yield stress of this composition is close to 7 Pa. A stress of 2 Pa is therefore clearly below this limit. Nevertheless, a monotonic increase in deformation is observed, which cannot be interpreted as anything other than steady-state flow. At this stress, the viscosity can only be estimated by order of magnitude and is at the order of 10^5^–10^6^ Pa s. This is a very high viscosity, and in this context it is useful to reconsider the possibility of flow below the yield point, which corresponds to the solid–liquid transition. It has been shown that the apparent viscosity of yielding samples depends on test duration [23] and has been regarded as an artifact caused by a long transition process [24]. This interpretation is often valid. However, the experimental data presented above suggest a slightly different view: the direct measurements demonstrate the possibility of steady-state flow below the yield point.

At the obtained high viscosity values, flow over the actual test duration leads to deformations at the order of 0.1 at 2 Pa. This means that most of the deformation remains plastic.

Additional information on the rheological behavior of the system is provided by the dependence of apparent viscosity on shear rate obtained in shear-rate-controlled deformation mode (Figure 13). At first glance, the curve appears to be a typical flow curve for a non-Newtonian fluid, but this is not the case. In the low-shear-rate region, the curve has a slope of −1 in logarithmic coordinates, corresponding to the reciprocal dependence of apparent viscosity on shear rate. This indicates a constant shear stress, as shown by the black dashed line in Figure 12a. The actual flow curve begins only at a shear rate of approximately 100 s^−1^. This is shown even more clearly in Figure 12b, where apparent viscosity is plotted as a function of shear stress. The transition from the gel-like state to the true flow curve is evident. The corresponding yield stress is σ_Y_ = 7 ± 1 Pa. The yield point can also be obtained by formally approximating the two branches in Figure 12a with power laws; the intersection of these dependencies also gives a stress of 7 Pa.

Clearly, the apparent “viscosity” values in the low-shear-rate region do not correspond to actual viscosity. These values are indeed apparent and may be considered artifacts. As shown in [23,24], this result is associated with the long transition process required to reach the yield point.

Stress-induced destruction of the yielding-liquid structure is clearly illustrated in Figure 13, which shows the results of up-and-down shear-rate scanning, as indicated by the arrows. The shear-rate scanning range covered the low-rate region, obviously below the corresponding yield point, and extended up to 40 s^−1^, which corresponds to a stress significantly higher than the yield point. Passing through the yield point during the decreasing shear-rate scan leads to a decrease in viscosity and to the disappearance of the constant-stress region, i.e., the yield point, although the character of the stress-shear-rate dependence changes somewhat near the yield-point region.

Thus, whereas the initial composition, gelatin plus sodium alginate (GSA), is a true gel, the introduction of 5% ZnO disrupts the gel structure and converts the medium into a yielding liquid with a clearly defined yield point.

The viscosity of this liquid after the yield point is exceeded is low, as is usually observed for yielding liquids. At low shear stresses, below the yield point, viscous flow with very high viscosity nevertheless exists, as shown in Figure 12. The possibility of very-high-viscosity flow in filled colloidal systems was discussed in early papers [25,26]. However, this possibility was later dismissed as an artifact [23]. According to the experimental data obtained here, flow in true gels is indeed impossible (Figure 10), but in yielding liquids such a possibility cannot be ruled out, as shown in Figure 12. The order of magnitude of the viscosity corresponding to flow in the “undisturbed structure” is close to that reported in [25,26].

**Figure 14:**
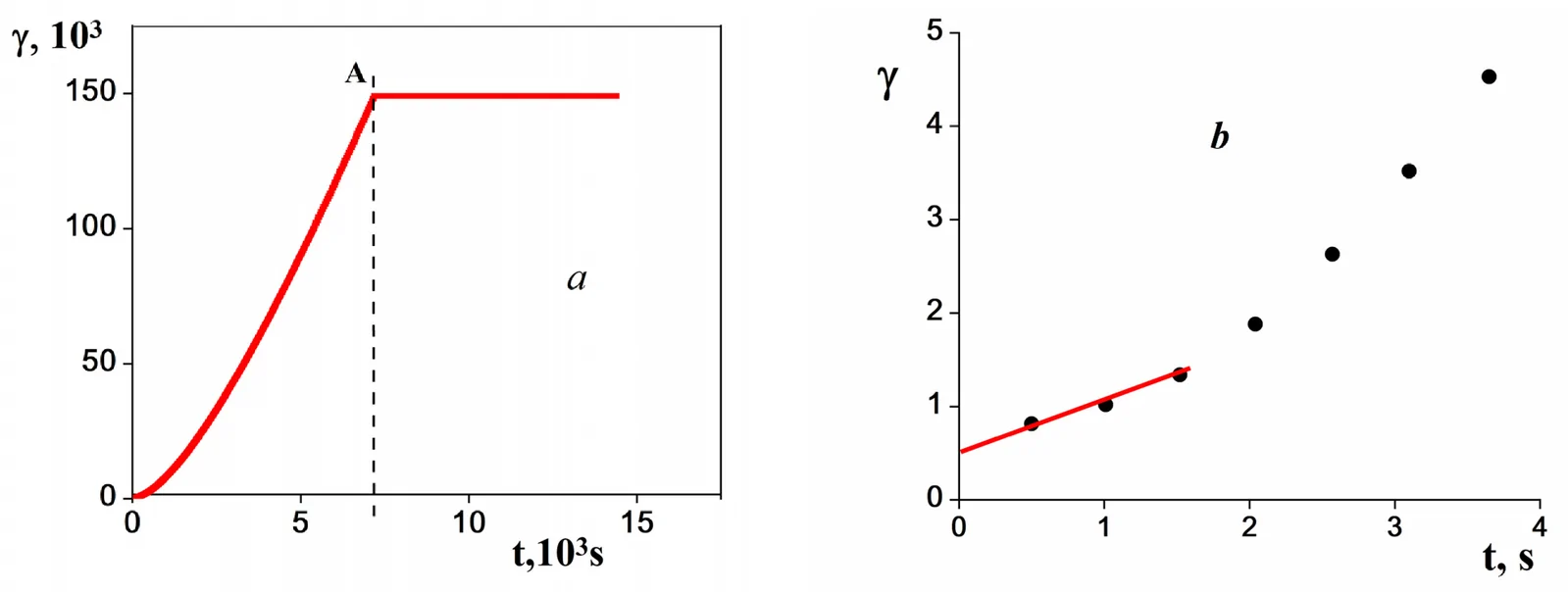
Development of deformation at σ = 20 Pa: (**a**) long-term loading; (**b**) initial time region for the GSA+5ZnO sample.

To clarify how the flow region is reached, we performed additional rheological measurements on the GSA+5ZnO sample at a higher stress, namely 20 Pa (Figure 14). The experiment consisted of measuring the deformation evolution first under a constant stress, in the 0A region, and then after the load was removed at point A. The long-term observation results are presented in Figure 14a, while the initial deformation region is shown separately on a different scale in Figure 14b.

The data in Figure 14a are clearly interpreted as viscous flow of a low-viscosity liquid. Thus, the flow corresponds to that of a liquid formed after the yield point has been exceeded. Estimation of the viscosity at the final stage of shear gives values in the range of 3 ± 1 Pa s, depending on the number of final points selected for calculation. This range is consistent with the data in Figure 14. Thus, viscous flow of a disrupted gel structure is indeed observed.

Figure 14b provides additional information on the behavior of the sample. At the initial moment of loading, a sharp jump in deformation is observed that is unrelated to flow. Its magnitude is considerable: 0.5 strain units correspond to 50%. At first glance, this jump may appear to be elastic deformation. However, at point A in Figure 14a, no rebound is observed, even when the scale is enlarged. This means that the initial jump should be attributed to plastic deformation, which is, of course, much smaller than the subsequent deformation caused by viscous flow.

**Figure 15:**
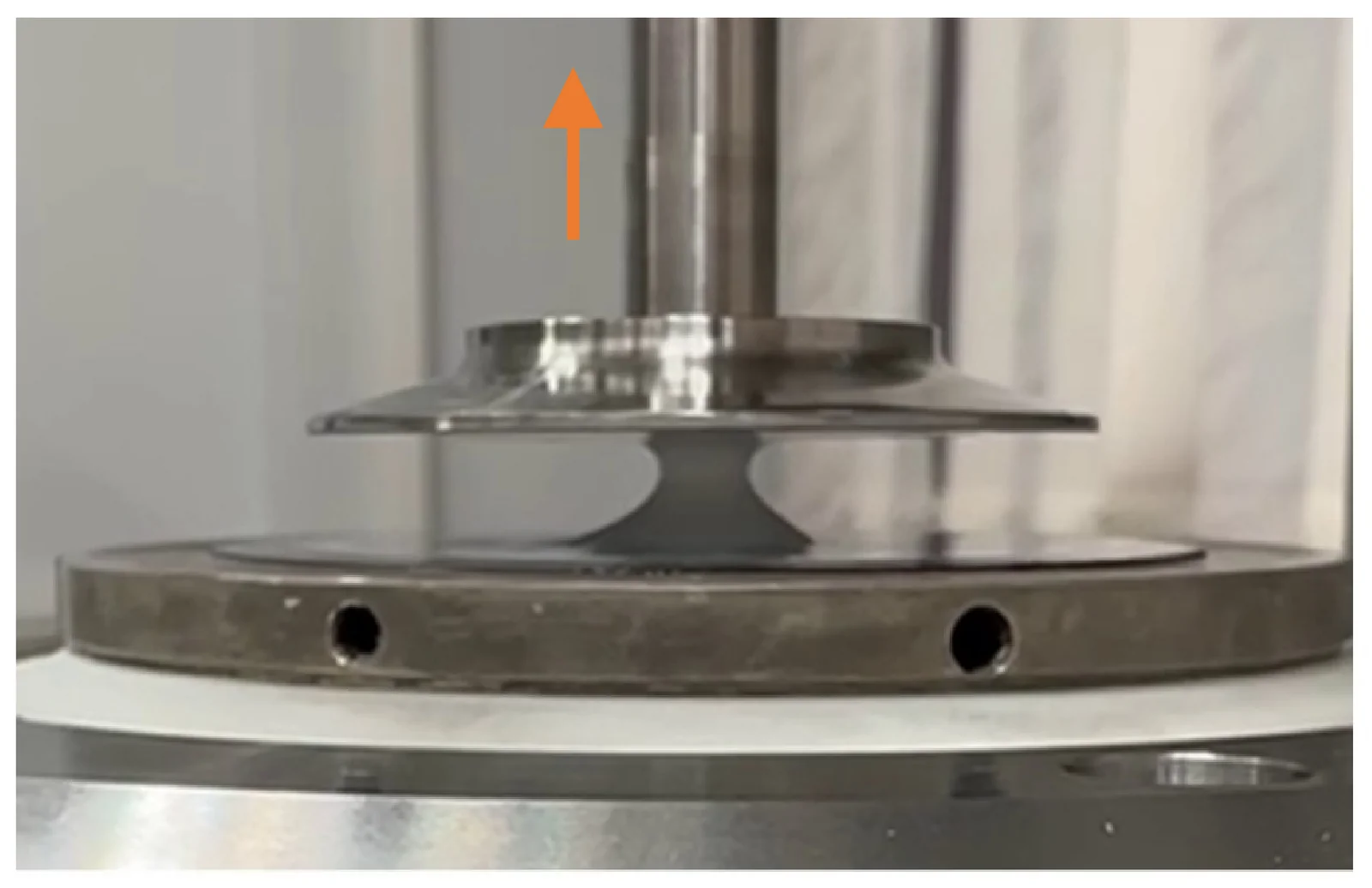
Pulling liquid from the surface of a viscous sample immediately before rupture of the filament. The arrow shows the direction of movement of the cone during the formation of a liquid bridge.

An additional visual experiment demonstrating that flow occurs in the viscometer gap rather than by wall slip was performed as follows. The upper cone was slowly raised above the surface, and video recording was used to monitor the stretching kinetics of the liquid filament connecting the surfaces until rupture. Figure 15 shows the frame immediately preceding filament rupture.

On the basis of the experimental data obtained, a yielding liquid can be considered, from a rheological perspective, as an elastic-plastic viscous fluid. Elastic-plastic behavior is observed below the yield point, whereas above the yield point the material transitions to a plastic-viscous state dominated by viscous flow.

### 2.5. FTIR Spectroscopy

In the discussion above, it was suggested that the physical origin of the observed effects is intermolecular interaction between the components, modified by the introduction of zinc oxide. A comparison of the IR spectra of the samples provides direct support for this interpretation.

**Figure 16:**
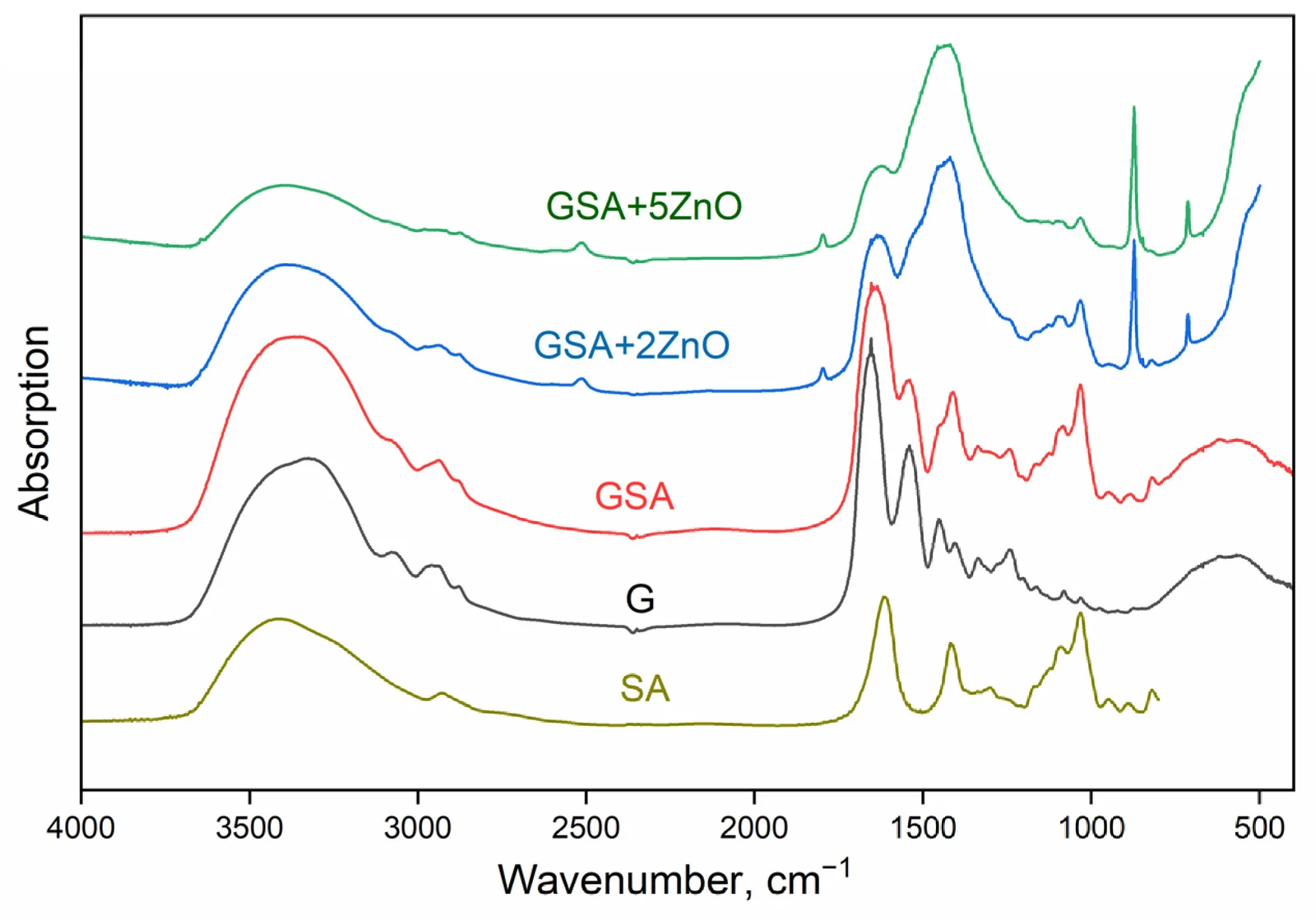
FTIR spectra of sodium alginate (SA), gelatin (G), GSA gel, and GSA compositions filled with ZnO nanoparticles at concentrations of 2% (GSA+2ZnO) and 5% (GSA+5ZnO).

Figure 16 shows the FTIR spectra of gelatin (G), sodium alginate (SA), the gelatin-sodium alginate gel (GSA), and this gel filled with zinc oxide nanoparticles at concentrations of 2% (GSA+2ZnO) and 5% (GSA+5ZnO).

**Figure 17:**
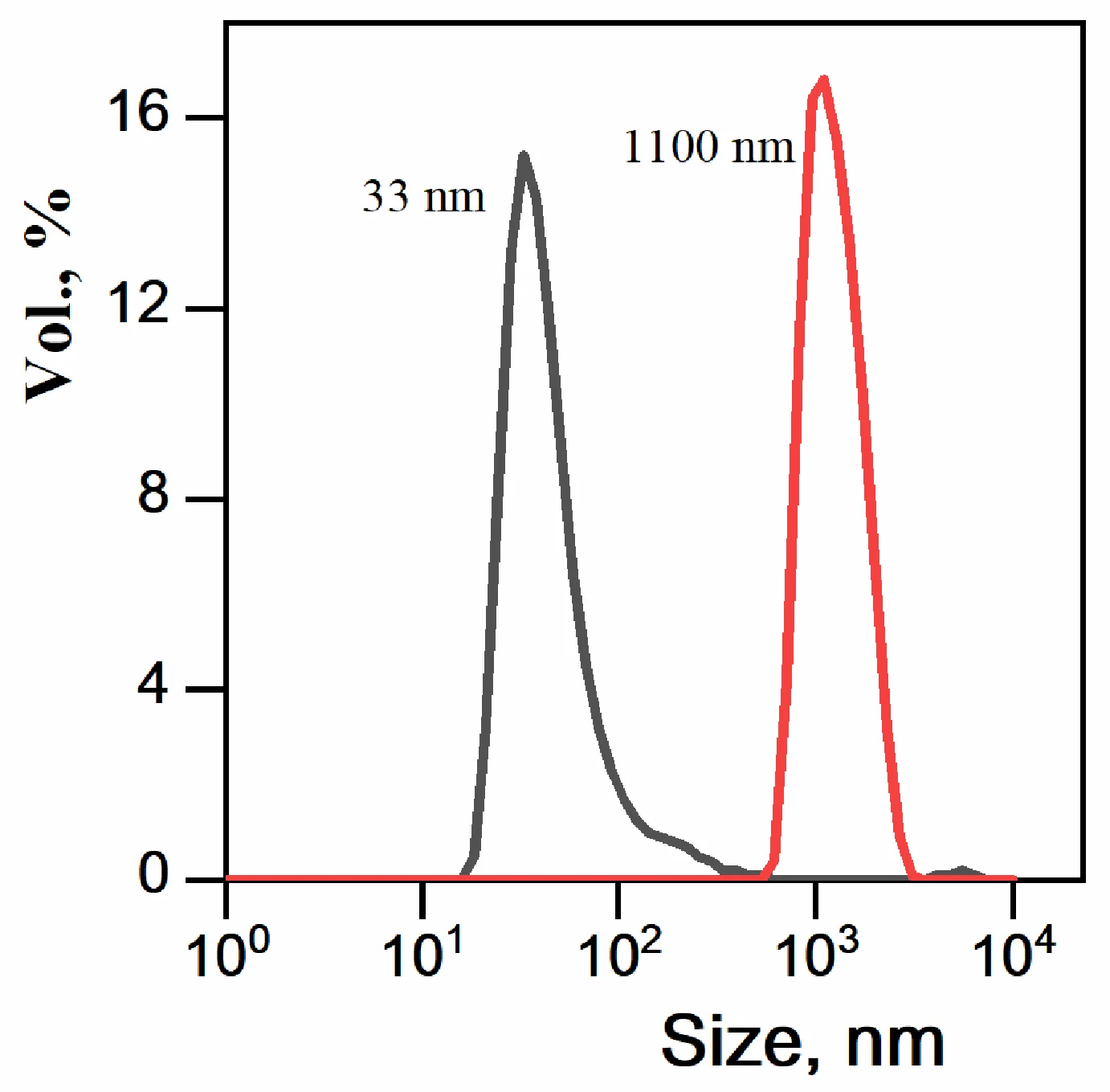
Distribution of ZnO particles measured by light scattering without surfactant (right curve) and with BYK-101 (left curve). The experiments were carried out by Dr. M.S. Kuzin.

The main characteristic bands in the FTIR spectrum of native gelatin (Figure 16, spectrum G) are a broad band with an absorption maximum at 3308 cm^−1^ (Amide A, stretching vibrations of N-H and O-H groups), and absorption bands at 1653 cm^−1^ (Amide I, stretching vibrations of C=O and C-N groups), 1545 cm^−1^ (Amide II, N-H and C-N vibrations), and 1239 cm^−1^ (Amide III, N-H and C-N stretching vibrations) [27]. The characteristic bands in the FTIR spectrum of native sodium alginate (Figure 17, spectrum SA) include a broad absorption band at 3420 cm^−1^ (O-H stretching vibrations) and bands at 1616 and 1418 cm^−1^ corresponding to the asymmetric and symmetric stretching vibrations of COO- groups, respectively [28].

Previous analysis of the FTIR spectra of gelatin-sodium alginate gels showed that gelatin-alginate complexes are formed through electrostatic interactions between the amino groups of gelatin (-NH_2_/-NH_3_^+^) and the carboxyl groups of sodium alginate (-COO^−^), as well as through hydrogen bonding [29].

The FTIR spectrum of the GSA gel (Figure 16, spectrum GSA) confirms this interpretation. The introduction of alginate into gelatin leads to a hypsochromic or blue shift in Amide A, from 3308 to 3363 cm^−1^, and to a bathochromic or red shift in Amide I, from 1653 to 1635 cm^−1^. Additional evidence for electrostatic interactions between gelatin and alginate is the shift in the symmetric stretching band of sodium alginate carboxylate groups (-COO^−^) to lower wavenumbers, from 1418 to 1408 cm^−1^. As a result, a network of hydrogen and ionic bonds is formed, producing a stronger gel network than that of gelatin alone and consequently changing the rheological properties of the gel [30,31].

The FTIR spectrum of the gel containing zinc oxide (ZnO) particles exhibits characteristic absorption bands associated with chemical-bond vibrations and structural features of the material [32]. These bands are located at 503 and 712 cm^−1^, corresponding to symmetric stretching vibrations of the Zn-O bond, and at 871 cm^−1^, corresponding to vibrations of Zn in tetrahedral coordination.

When ZnO nanoparticles are introduced into the gel, shifts and broadening of the characteristic bands are observed in the IR spectrum compared with the spectrum of the gel without nanoparticles. Amide I shifts to lower wavenumbers, from 1635 to 1626 cm^−1^ for GSA+2ZnO and to 1616 cm^−1^ for GSA+5ZnO. At the same time, its intensity decreases with increasing nanoparticle concentration. Broadening and weakening of the Amide A band (O-H/N-H) are also observed, and these effects become more pronounced as the nanoparticle concentration increases. The position of the Amide A maximum shifts to higher wavenumbers, from 3363 cm^−1^ to 3389 cm^−1^ for GSA+2ZnO and to 3403 cm^−1^ for GSA+5ZnO. The absorption bands in the range 1580–1300 cm^−1^, corresponding to gelatin Amide II and alginate COO^−^ groups, overlap, which also indirectly indicates band broadening in the presence of zinc oxide.

Changes in the FTIR spectrum of the nanoparticle-filled GSA gel indicate that the carboxyl groups of alginate and the carbonyl and amine groups of gelatin coordinate with the surface of ZnO nanoparticles; this interaction becomes more pronounced as the nanoparticle concentration increases. Thus, zinc oxide shields charged groups of the biopolymers and competes for binding sites, reducing direct contact between gelatin and alginate and weakening or disrupting electrostatic interactions and hydrogen bonds. Consequently, the structure of the biopolymer gel changes and the density of its structural network decreases, as discussed above (Figure 2, Figure 3 and Figure 6).

## 3. Conclusions

The study of the rheological properties of a hydrogel and the yielding liquids derived from it, considered as typical examples of soft matter. The definition of gel-state based on mechanical testing and measurement of elasticity and stress relaxation was discussed in an earlier publication [33]. In this study it has been shown that irreversible plastic deformations occur when these objects are subjected to shear deformation under constant stress. Their characteristic feature is that they depend on stress but, unlike flow, are independent of time. It is noteworthy that, unlike plastic deformation in solid mechanics, where plastic deformation occurs only after a certain elastic-deformation threshold is reached, in the systems considered here plastic deformation develops in parallel with elastic deformation. As expected, the true gel does not flow. In contrast, in the gel-like state of yielding liquids, at stresses below the yield point, a monotonic increase in deformation with time is observed in addition to elastic and plastic deformation. This behavior is associated with viscous flow at very high viscosity, at the order of 10^5^–10^6^ Pa s. After the yield point is exceeded, ordinary liquid flow with a viscosity of approximately 3 Pa s occurs. However, noticeable plastic deformation is still observed at the onset of deformation. The proposed mechanism of plastic deformation is related to shear-induced reorganization of the physical-bond network. This interpretation is supported by direct structural observations and IR-spectroscopy data.

Plasticity in mechanics is an independent phenomenon that is not described by theories of elasticity or fluid dynamics. Unlike elasticity, plasticity is the development of irreversible deformations and the manifestation of the nonlinear properties of a medium. At the same time, plasticity is distinguished by the absence of a time dependence of deformation at a given stress on flow, at which deformations grow nonlinearly. This understanding of plasticity was repeatedly mentioned both in the introduction and in the discussion of experimental data.

As shown in the study, gels (like some other objects) are capable of plastic deformation. Consequently, constructing any models of the behavior of these materials is impossible without incorporating the concept of plasticity as an essential attribute of their behavior. The existence of plastic deformation in soft matter has not been taken into account in modern rheological models of complex media [34,35]. This phenomenon cannot be described by a combination of traditional mechanical elements such as springs, dashpots, and surface-friction sliders, which are often used to describe the behavior of yielding liquids. Instead, it should be considered with the addition of plasticity theory.

Finally, it is worth noting the use of plastic deformation in technological practice. Shaping products through plastic deformation is common for soft matter, such as highly concentrated suspensions, compositions used for 3D printing and powder casting [36,37,38], and culinary products, the shape of which is formed by the deforming of gel-like compositions. A huge area of application of plastic deformations is associated with the processing of soft metals (stamping, forging, drawing).

## 4. Materials and Methods

### 4.1. Materials

Two types of samples were used in the experiments. The first was a gelatin-sodium alginate gel consisting of 1% gelatin (G) in deionized water with sodium alginate (SA) added at a concentration of 0.8%. The second was the same gel modified by the introduction of 2 or 5 vol.% nanosized ZnO particles, denoted as GSA+2ZnO and GSA+5ZnO, respectively. Such filled hydrogels are of particular interest for two reasons. First, solid nanoparticles may form an independent structure within the gel or interact with macromolecules, thereby affecting the rheological properties. Second, such systems are of technological interest for applications in photocatalytic dye degradation [19].

To determine the particle size distribution of ZnO, a suspension was prepared in distilled water at a solid-phase mass concentration of 0.5%. The suspensions were stabilized by adding 0.1% Disperbyk-101, a commercial product manufactured by BYK-Chemie GmbH, Wesel, Germany. The dispersant was not used in the preparation of the samples studied rheologically.

All samples were mechanically processed using an ULTRA-TURRAX IKA T10 Basic rotor-stator disperser (Staufen, Germany) at 30,000 rpm for 5 min. The particle size distribution was determined by dynamic light scattering using a Zetasizer ZS instrument (Malvern, UK) in backscattering mode (173°) at 25 °C. The averaged distributions obtained from several measurements are shown in Figure 17. The use of the disperser made it possible to obtain a nanofiller with a relatively narrow size distribution, as shown by the left-hand curve in Figure 1.

The GSA sample was a complex gel composed of gelatin (C = 1.0%) and sodium alginate (C = 0.8%). Gelatin and sodium alginate solutions were prepared separately by pre-soaking the calculated amounts of gelatin and sodium alginate for 1 and 3 h, respectively, followed by dissolution at 45 and 60 °C. The required volumes of the initial solutions were mixed and kept at 4 °C for 16 h to form the gel.

The second type of sample, GSA+ZnO, was a complex gel filled with zinc oxide nanoparticles at concentrations of 2 and 5 vol.% (GSA+2ZnO and GSA+5ZnO, respectively). The calculated amount of zinc oxide was added to the gelatin-sodium alginate solution prepared as described above. To ensure uniform nanoparticle distribution, the mixture was sonicated for 30 s with external cooling of the vessel in an ice bath. The resulting mixture was kept at 4 °C for 16 h to form samples for further investigation.

### 4.2. Methods

#### 4.2.1. Rheology

Rheological measurements were performed using a Physica MCR 302 rheometer (Anton Paar GmbH, Graz, Austria) equipped with a cone-and-plate measuring system (plate diameter 50 mm; cone angle 1°; gap 0.100 mm).

The rheological characterization consisted of two series of measurements. In the first series, the samples were loaded at a constant shear stress for 35 min, while the increase in deformation under stress was recorded. The load was then removed, and reverse deformation was monitored. These experiments were performed over a stress range of 0.5–10 Pa. The second series consisted of long-term loading for 5 h at constant stresses of 2 or 10 Pa. The deformation evolution was recorded after 2, 3, 4, and 5 h, followed by measurement of elastic recovery. All measurements were carried out at 4.00 ± 0.03 °C. Sample temperature was controlled using Peltier elements (P-PTD200/GL) (Neuried, Bayern). The procedure used to process the experimental data is described and discussed in the next section.

When discussing the experimental data presented below, it should be borne in mind that the properties of the materials considered are highly sensitive to minor details of sample preparation. As a result, repeated experiments showed considerable scatter, reaching approximately 10%. Therefore, the general character of the observed deformations, rather than specific numerical values, is most important for identifying their physical nature.

#### 4.2.2. Scanning Electron Microscopy

The microstructure of the samples was studied by scanning electron microscopy (SEM) using a Merlin field-emission microscope (Carl Zeiss, Oberkochen, Germany) operated at an accelerating voltage of 5 kV. For analysis, the samples were frozen in liquid nitrogen under vacuum. A Labfreez FD-10-MR freeze-dryer (Labfreez Co., Ltd., Beijing, China) with a condenser temperature below −55 °C and an ultimate residual pressure of 5 Pa was used. Sections of the frozen samples were coated with gold/palladium (80/20).

Quantitative analysis of the sample microstructure was performed using MountainsLab software V11.0 (Digital Surf, Besançon, France).

#### 4.2.3. FTIR Spectroscopy

GSA and GSA+ZnO hydrogel samples were mixed with KBr powder at a 1:1 *w*/*w* ratio and freeze-dried at −50 °C and 9.8 Pa for 6 h using a FreeZone 6 L freeze dryer (Labconco Corp., Kansas City, MO, USA). The samples were then dried at 60 ± 1 °C to remove residual moisture. Finally, 150 mg of the highly dispersed powder was pressed into a pellet at 650 kgf/cm^2^.

FTIR absorption spectra were recorded using a Shimadzu IR Tracer-100 FTIR spectrometer (Shimadzu Corp., Kyoto, Japan) over the wavenumber range from 4000 to 400 cm^−1^. The number of scans was 250, and the resolution was 4 cm^−1^. The obtained FTIR spectra were digitally processed using OriginPro software 9.0 (Northampton, MA, USA) to refine peak positions and correct the baseline.

## Data Availability

The data that support the findings of this study are available from the corresponding author upon reasonable request.
